# Response of soil fungal communities to long-term soil mercury pollution

**DOI:** 10.3389/fmicb.2026.1827403

**Published:** 2026-06-10

**Authors:** Jianxiong Du, Jianfeng Li, Shuqing Zhang, Yuxiang Ren

**Affiliations:** 1 School of Management Science and Engineering, Guizhou University of Finance and Economics, Guiyang, China; 2 Key Laboratory of Biological Resources Exploitation and Utilization in Universities of Guizhou, Guizhou Education University, Guiyang, China; 3 School of Foreign Languages, Guizhou Normal University, Guiyang, China

**Keywords:** class, fungi, mercury, phylum, relative abundance

## Abstract

Mercury naturally occurs in soil and can accumulate in high concentrations because of various human activities. In order to further explore the effect of long-term mercury contamination of soil near a mercury mining area (Tongren, Guizhou Province, China) on soil fungal communities. Soil samples (mercury content: SMO20 > SMO2 > SMO650 > SMO30 > SMO500) were collected from five locations at distances of 2 m, 20 m, 30 m, 500 m, and 650 m, respectively, from the only sewage outlet of a mercury mining area (Guizhou, China). The soil microbial DNA was extracted from each soil sample and sequenced via high-throughput sequencing technology. The sequencing results indicated that, at both the phylum and class levels, the soil fungal community diversity of SMO2 and SMO30 was greater than that of SMO20, SMO500, and SMO650. The soil fungal community structure analysis revealed common and unique dominant fungal communities within the soil sample groups at both the phylum and class levels. Redundancy analysis (RDA) of relationships between soil fungal community structure and soil environmental factors (pH, EC, available N, P, K, and mercury content) revealed that mercury was the most influential factor. The survival of high-abundance fungal community taxa is strong evidence of the fungal community’s high adaptability to long-term soil mercury contamination. The results of this study provide a scientific basis for further studies on the mechanism of mercury tolerance in soil fungi under long-term mercury stress.

## 1 Introduction

The rapid advancement of global industrialization has led to increasingly severe soil pollution worldwide, with frequent reports of heavy metal contamination in recent years (Mputu et al., 2025; Alo et al., 2025; Cora et al., 2025). Once introduced into the soil, heavy metals can migrate, undergo transformations, and decompose, leading to adverse effects such as degradation of soil quality and instability of soil ecosystems (Qian et al., 2025; Adewumi and Ogundele, 2024; Adedeji et al., 2020).

Mercury, a toxic heavy metal, has led to widespread soil pollution worldwide in recent years, posing significant threats to global ecological security (Uwiringiyimana et al., 2023; Melissa et al., 2023). Primary sources of soil mercury contamination include industrial emissions, mercury mining, mineral processing, and smelting activities (Xu et al., 2015; Liu et al., 2021). Mercury disrupts the internal soil ecosystem, and gaseous or particulate mercury released into the atmosphere reenters the soil system through dry and wet deposition, further exacerbating contamination (Rytuba, 2003). Mercury and its compounds are toxic; organic mercury, especially methylmercury, is more toxic than inorganic mercury. Mercury can also undergo transformations between different chemical valences. When soil mercury concentrations exceed certain thresholds, plant growth, animal survival, and microbial stability are severely affected. Moreover, through food chain accumulation and biomagnification, mercury poses considerable risks to habitat safety (Zhang et al., 2025; Senila et al., 2025). Soil microorganisms are a vital and highly active component of the soil ecosystem, playing a key role in the entire process of soil development; they significantly enhance soil fertility, facilitate the cycle of carbon, nitrogen, sulfur, phosphorus, and other elements, purify pollutants, and maintain ecosystem balance (Liu et al., 2024; Gliesch et al., 2025). The soil microbial community, including bacteria, fungi, actinomycetes, and other groups, is critical to maintaining the stability and functioning of the soil ecosystem. Previous research has shown that soil microorganisms are more sensitive to heavy metals than animals or plants (Lin et al., 2025; Shalini and Charu, 2018; Mahbub et al., 2017). Consequently, they are regarded as highly promising indicators for assessing soil quality and serve as practical, sensitive metrics for evaluating soil-related impacts on human health (Lin et al., 2025). Heavy metal exposure induces chemical, physiological, and morphological changes in microbial systems, affecting processes such as protein synthesis, lipid metabolism, basal respiration, and energy exchange (Lajayer et al., 2017; Wang et al., 2020). Currently, researchers commonly evaluate the effects of heavy metals on microbes by analyzing microbial biomass, activity, and community structure (Cora et al., 2025). Mercury in soil can inhibit the growth of fungi, actinomycetes, and bacteria, and strongly affect microbial activity and community structure (Salam et al., 2019). Little attention has been paid to the response of soil fungal communities to long-term mercury contamination. Specifically, the variation in community diversity and structure along a gradient of mercury concentrations is poorly characterized. Consequently, elucidating the response traits of these communities to prolonged mercury exposure is paramount for informing and advancing bioremediation approaches.

In this study, topsoil samples (0–20 cm depth) were collected at various distances from a single sewage outlet near an abandoned mercury mine in Tongren, Guizhou Province, China. High-throughput sequencing was used to examine how mercury contamination affects the diversity and structure of the soil fungal community. We expect that our results will provide meaningful insights into mercury’s impact on fungal communities and support future exploration of mercury-resistant fungi and plants for remediating contaminated soils.

## 2 Methods

### 2.1 A brief introduction to the study site

**Figure 1:**
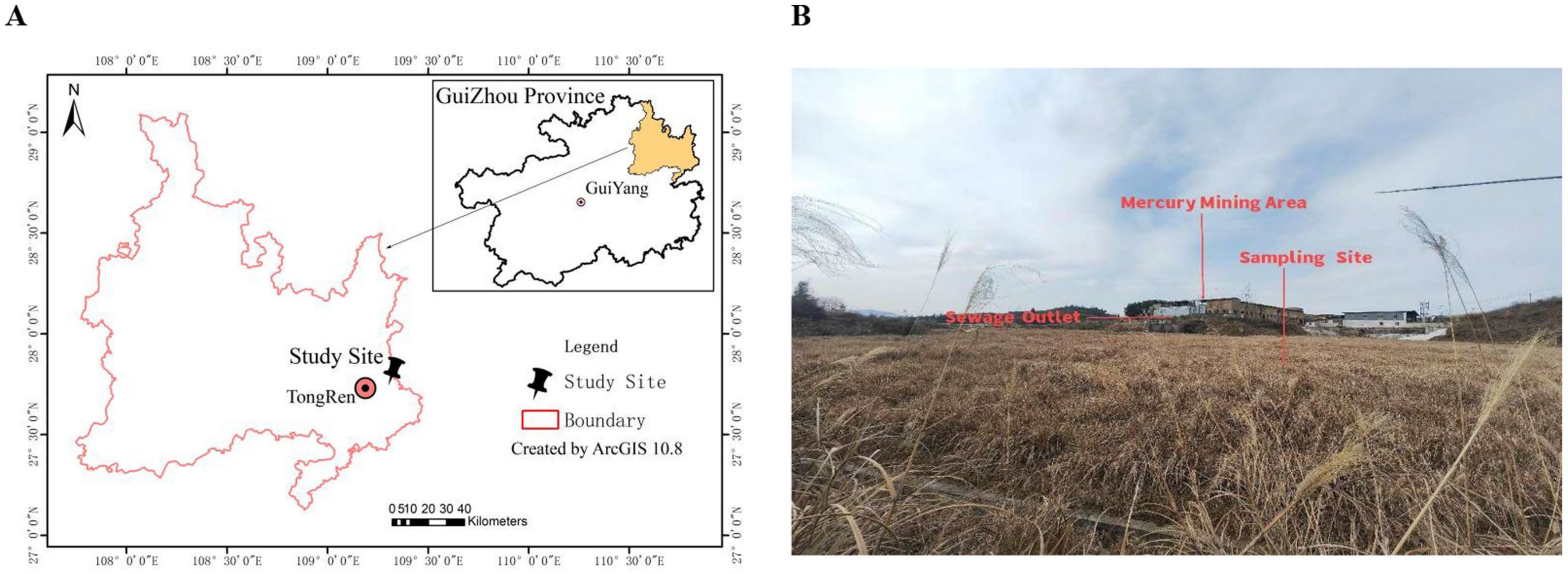
**(A)** The map of the study site (modified from Du et al., 2023, 2025). **(B)** The soil sampling site.

The study area (109°07′–109°24′E; 27°24′–27°38’ N) is situated in the northeastern part of Tongren City, Guizhou Province, China (Figures 1A,B). This region historically contained the highest mercury reserves in China and across Asia. The landscape is characterized by low mountains, hills, and valleys, with brown soil as the predominant soil type. The region experiences an annual average temperature of approximately 13–14 °C. Due to prolonged overexploitation and the subsequent depletion of mercury resources, the local government has closed the mercury mines (Liu, 2022). A map of the study site (Figure 1A) was generated using ArcGIS software (version 10.8, USA).

### 2.2 Soil sampling

Soil samples were collected from the topsoil layer (0–20 cm depth) near an abandoned mercury mine in Tongren, Guizhou Province, China. The sampling sites were radially distributed from a central sewage outlet. Based on the distance from the outlet, samples were categorized into five treatment groups: SMO2, SMO20, SMO30, SMO500, and SMO650. Each group included four replicates. Specifically, the SMO2 group (replicates labeled MSO1, MSO2, MSO3, MSO4) was sampled at 2 m radius; SMO20 (CMSO21, CMSO22, CMSO23, CMSO24) at 20 m; SMO30 (GMSO301, GMSO302, GMSO303, GMSO304) at 30 m; SMO500 (GMSO500, GMSO501, GMSO502, GMSO503) at 500 m; and SMO650 (GMSO651, GMSO652, GMSO653, GMSO654) at 650 m. The four replicates within each group were collected at distinct intervals along the same radial distance.

After processing, all soil samples were crushed to remove impurities and then sieved through a 1 mm mesh. Each sample was divided into two aliquots: one for soil property analysis (cured naturally at 25–35 °C and 20–60% relative humidity) and the other for microbial DNA extraction (stored at −20 °C).

### 2.3 Soil properties

The mercury content in the soil samples was measured using a cold-atom absorption mercury analyzer (F732–V, China) (Qian, 2018), which has a detection limit of 0.05 μg/L. Soil electrical conductivity (EC, μS/cm) and pH were determined with a portable conductivity meter (EC–450, USA) and a pH meter (HANNA HI98107, Italy), respectively. The available nitrogen (N) content was analyzed by the alkaline hydrolysis diffusion method (Yang et al., 2022); available phosphorus (P) was measured by the molybdenum antimony ascorbic acid colorimetric method (Yang et al., 2022); and available potassium (K) was determined by flame photometry (Yang et al., 2022). The properties of all soil samples are summarized in Table 1.

**Table 1:** The properties of the five different groups of soil samples (modified from Du et al., 2023, 2025).

Groups of soil samples		Distance from sewage outlet(m)	Available N (mg/kg)		Available P (mg/kg)		Available K (mg/kg)		EC (μs/cm)	pH	Mercury (mg/kg)	
SMO2	MSO1	0.13	9.06	9.04 ± 0.06	11.14	11.06 ± 0.07	29.21	29.24 ± 1.21	122	7.5	147.04	140.45 ± 15.56
	MSO2	0.16	9.11		11.02		30.06		124	7.5	139.23	
	MSO3	0.18	8.98		11.08		30.14		125	7.5	119.51	
	MSO4	0.22	9.02		10.99		27.54		123	7.5	156.03	
SMO20	CMSO1	21.2	8.07	8.03 ± 0.07	10.34	10.43 ± 0.41	27.24	27.70 ± 0.49	112	8.0	167.14	152.44 ± 22.22
	CMSO2	20.10	8.01		10.13		27.31		114	8.0	139.25	
	CMSO3	19.60	7.94		10.22		28.07		110	8.0	128.33	
	CMSO4	20.32	8.11		11.04		28.16		113	8.5	175.02	
SMO30	GMSO301	30.50	8.97	8.63 ± 0.46	12.03	11.69 ± 0.45	30.04	29.29 ± 1.16	119	7.5	69.15	63.48 ± 11.18
	GMSO302	30.20	9.07		12.10		30.12		122	8.0	72.31	
	GMSO303	29.80	8.31		11.43		29.36		121	7.5	47.29	
	GMSO304	30.10	8.16		11.19		27.62		126	7.5	65.18	
SMO500	GMSO500	500.20	8.09	8.37 ± 0.44	10.21	10.67 ± 0.47	30.02	29.16 ± 1.03	114	8.5	61.51	59.77 ± 11.34
	GMSO501	500	8.21		10.32		28.22		123	8.5	45.42	
	GMSO502	500.10	8.15		11.03		28.31		116	8.5	73.06	
	GMSO503	499.80	9.02		11.11		30.07		126	8.5	59.10	
SMO650	GMSO651	650.30	9.11	8.88 ± 0.34	11.39	11.90 ± 0.34	29.11	28.57 ± 0.80	123	6.5	96.32	109.44 ± 12.35
	GMSO652	650.20	8.98		12.01		28.52		120	7.0	112.04	
	GMSO653	650.40	9.05		12.14		29.19		126	6.5	125.27	
	GMSO654	650.10	8.37		12.06		27.45		124	7.0	104.13	

The data are presented as the means ± standard deviations.

The contents of available nitrogen (AN), available phosphorus (AP), and available potassium (AK) in the soil samples ranged from 8.03 to 9.04 mg/kg, 10.43 to 11.90 mg/kg, and 27.70 to 29.29 mg/kg, respectively. Soil electrical conductivity (EC) values varied between 112 and 126 μs/cm, while soil pH ranged between 6.5 and 8.5. The mercury content across different sampling groups followed the order: SMO20 > SMO2 > SMO650 > SMO30 > SMO500. Specifically, the mercury content in groups SMO2, SMO20, and SMO650 was significantly higher than that in SMO30 and SMO500. In healthy soil, the acceptable mercury level is 0.01–0.3 mg/kg, as referenced by WHO. In this study, the mercury content of soil samples (59.77–152.44 mg/kg) was significantly higher than the soil environmental risk screening value (>1.0 mg/kg, GB 15618-2018, China), indicating severe mercury pollution.

### 2.4 DNA extraction

Total community genomic DNA was extracted from the samples using the E.Z.N.A.™ Mag-Bind Soil DNA Kit (Omega, M5635-02, USA) according to the manufacturer’s instructions (Omega Bio-tek, 2018). The concentration of the extracted DNA was then measured with a Qubit 4.0 fluorometer (Thermo Fisher Scientific, USA) to obtain an accurate quantification of high-quality genomic DNA for downstream applications.

### 2.5 ITS rRNA gene amplification via PCR

The ITS1-ITS2 hypervariable region of the fungal ITS rRNA gene was targeted for analysis. Following DNA extraction, polymerase chain reaction (PCR) amplification was performed using a 2 × Hieff^®^ Robust PCR Master Mix (Yeasen, 10105ES03, China) (Yeasen Biotechnology Shanghai Co., Ltd., 2020). Amplification was carried out with the universal fungal ITS primers ITS1-F (forward: CTTGGTCATTTAGAGGAAGTAA) and ITS2 (reverse: GCTGCGTTCTTCATCGATGC), both of which were PAGE-purified.

### 2.6 ITS gene library preparation, quantification, and sequencing

Amplicon products were purified using Hieff NGS^™^ DNA Selection Beads (Yeasen, 10105ES03, China) to remove primer dimers and free primers. The purified samples were sent to Sangon Biotech (Shanghai) for library construction with universal Illumina adapters and indexes. Prior to sequencing, the DNA concentration of each PCR product was quantified using the Qubit^®^ 4.0 Green double-stranded DNA assay, and quality was assessed with an Agilent 2100 Bioanalyzer. Based on sequencing coverage requirements, all libraries were pooled and sequenced in a single run. Equimolar mixing of amplicons was performed according to their measured concentrations. Sequencing was performed on the Illumina MiSeq PE300 platform (Illumina, USA) according to the manufacturer’s recommended protocol.

### 2.7 Sequence processing, OTU clustering, representative tag alignment, and biological classification

Following sequencing, Illumina paired—end reads were merged using PEAR software (v0.9.8) based on sequence overlap. The resulting FASTQ files were processed to generate corresponding FASTA files and quality (QUAL) files for subsequent analysis. USEARCH software (v11.0.667) was then used to cluster high-quality sequences into operational taxonomic units (OTUs) at a ≥ 97% similarity threshold. Sequences were assigned to respective samples according to their OTU classifications, after which chimeric sequences and singleton OTUs (represented by only a single read) were removed. The most abundant sequence within each OTU cluster was selected as the representative sequence. Taxonomic classification of fungal OTUs was performed by BLAST alignment against the UNITE fungal ITS database.

### 2.8 Statistical analysis

Alpha diversity indices (Simpson and Shannon) were calculated based on OTU richness using Mothur (v3.8.31). Differences in fungal community alpha diversity among multiple groups were assessed using ANOVA, and redundancy analysis (RDA) was conducted with the vegan package (v2.5–6) in R.

## 3 Results

### 3.1 Soil fungal community diversity at the phylum level

**Figure 2:**
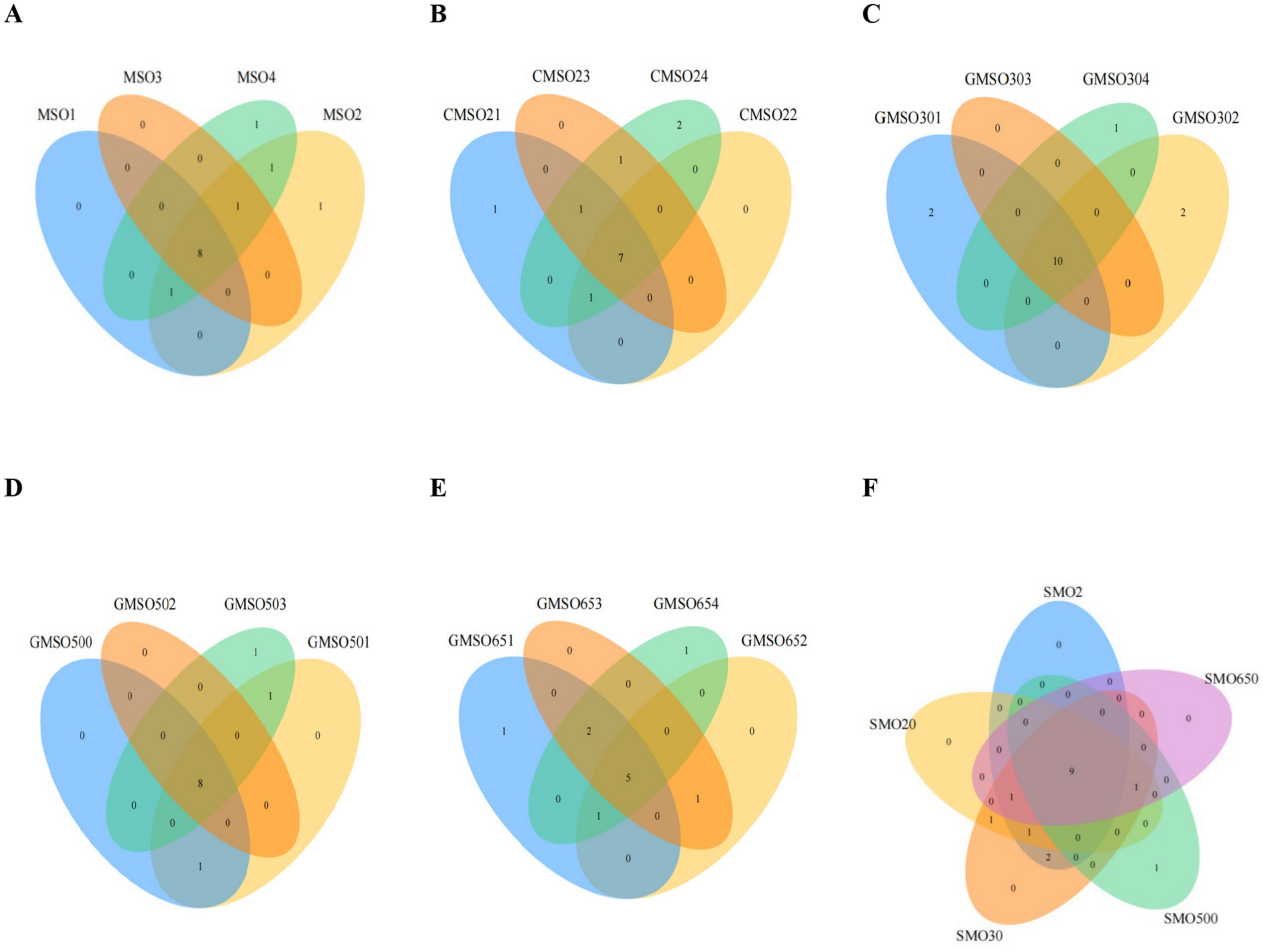
**(A)** The operational taxonomic units (OTUs) number of the SMO2 group at the phylum level. **(B)** The number of OTUs in the SMO20 group at the phylum level. **(C)** The number of OTUs in the SMO30 group at the phylum level. **(D)** The number of OTUs in the SMO500 group at the phylum level. **(E)** The number of OTUs in the SMO650 group at the phylum level. **(F)** The OTUs are distributed across five groups at the phylum level.

At the phylum level, Venn diagrams (Figures 2A–F) were generated using the VennDiagram package in R following OTU clustering of sample sequences at a 97% similarity threshold. The numbers of OTUs identified were 13 in SMO2 group (Figure 2A), 13 in SMO20 (Figure 2B), 15 in SMO30 (Figure 2C), 11 in SMO500 (Figure 2D), and 11 in SMO650 (Figure 2E). These results demonstrated that the OTU richness in SMO2, SMO20, and SMO30 groups was greater than that in SMO500 and SMO650 groups. Furthermore, within-group analysis revealed variations in OTU numbers among the four replicates of each sample group, with notable differences observed particularly in the SMO20 and SMO30 groups.

The OTU distribution across the five treatment groups is summarized in Figure 2F. A total of 16 OTUs were identified, 9 of which were shared by all groups. The SMO500 group contained 1 unique OTU, while the other four groups had none, suggesting that intergroup differences in OTU numbers at the phylum level were not significant.

### 3.2 Soil fungal community diversity at the class level

**Figure 3:**
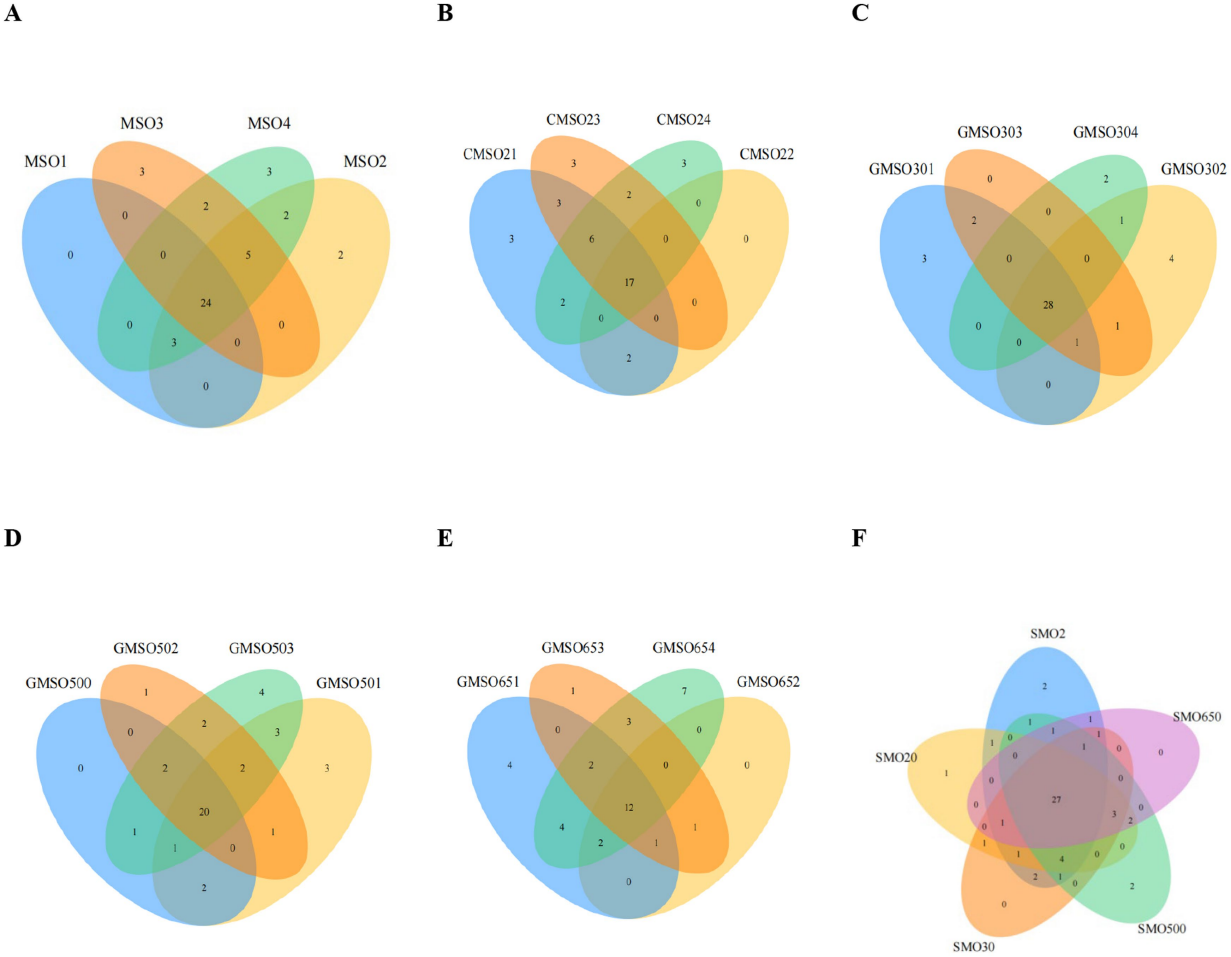
**(A)** The operational taxonomic units (OTUs) number of the SMO2 group at the class level. **(B)** The number of OTUs in the SMO20 group at the class level. **(C)** The OTUs number of SMO30 group at the class level. **(D)** The OTUs number of SMO500 group at the class level. **(E)** The OTUs number of SMO650 group at the class level. **(F)** The OTUs are distributed across five groups at the class level.

At the class level, Venn diagrams (Figures 3A–F) were generated using the VennDiagram package in R following OTU clustering of sample sequences at a 97% similarity threshold. The numbers of OTUs identified were 44 in SMO2 group (Figure 3A), 41 in SMO20 (Figure 3B), 42 in SMO30 (Figure 3C), 42 in SMO500 (Figure 3D), and 37 in SMO650 (Figure 3E). These results demonstrated that the OTU richness in SMO2, SMO20, SMO30, and SMO500 groups was greater than that in SMO650 group. Additionally, the number of OTUs common to all replicates within each group was 24 in SMO2, 17 in SMO20, 28 in SMO30, 20 in SMO500, and 12 in SMO650. Within-group comparisons revealed significant variation in OTU counts across the four replicates within each treatment group, with particularly pronounced differences in the SMO20 and SMO650 groups.

Analysis of OTU numbers at the class level revealed significant differences among the five groups (Figure 3F). Of the 53 OTUs identified, 27 were shared by all groups. The unique OTUs were distributed as follows: 2 in SMO2, 1 in SMO20, 2 in SMO500, and none in either SMO30 or SMO650.

### 3.3 Alpha diversity analysis

**Figure 4:**
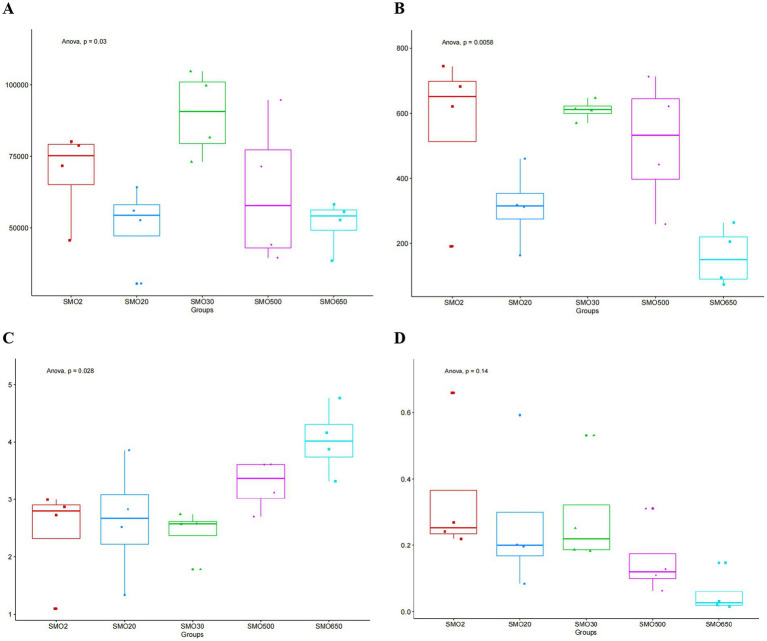
**(A)** The reads of five different groups. **(B)** The operational taxonomic units (OTUs) of five different groups. **(C)** The Shannon index of five different groups. **(D)** The Simpson index of five different groups.

The alpha diversity indices of the fungal communities in five groups were shown in Figures 4A–D. The read counts and OTU numbers were highest in the SMO30 group, followed by the SMO2, SMO500, SMO650, and SMO20 (Figures 4A,B). In contrast, the Shannon index was highest in SMO650, followed by SMO500, SMO2, SMO20, and SMO30 (Figure 4C), while the Simpson index ranked SMO2 highest, followed by SMO30, SMO20, SMO500, and SMO650 (Figure 4D). Significant intergroup differences were observed for read counts, OTU numbers, and the Shannon index (*p* < 0.05, ANOVA), indicating that SMO2 and SMO30 groups generally possessed greater alpha diversity than SMO20, SMO650, and SMO500 groups.

### 3.4 The soil fungal community structure at the phylum level

**Figure 5:**
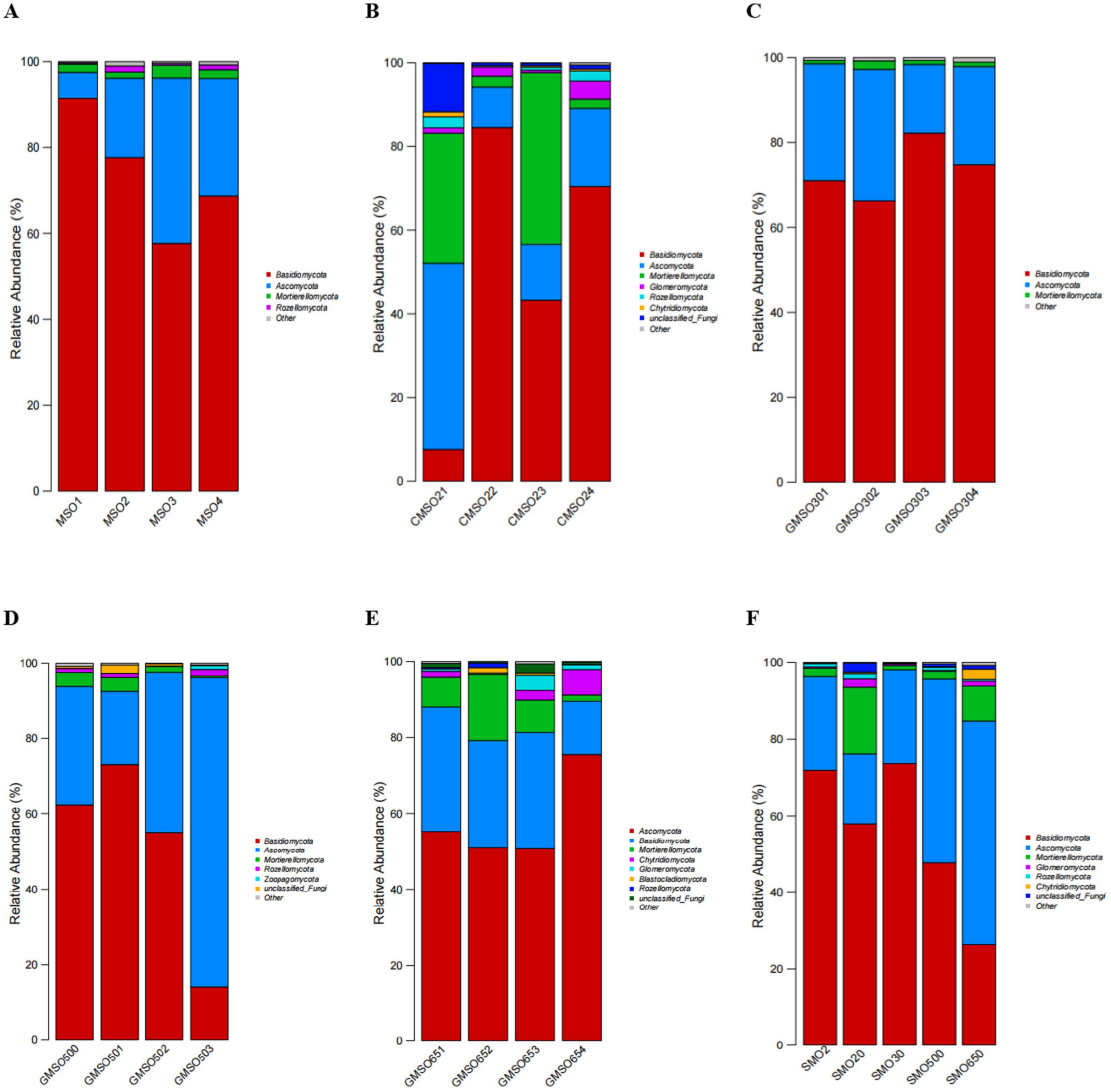
**(A)** The soil fungal community relative abundance of the SMO2 group at the phylum level. **(B)** The soil fungal community relative abundance of the SMO20 group at the phylum level. **(C)** The soil fungal community relative abundance of the SMO30 group at the phylum level. **(D)** The soil fungal community relative abundance of the SMO500 group at the phylum level. **(E)** The soil fungal community relative abundance of the SMO650 group at the phylum level. **(F)** The soil fungal community relative abundances of five different groups at the phylum level.

At the phylum level, Figure 5A displays the relative abundances of soil fungal communities in the SMO2 group. Extremely high relative abundances were observed for *Basidiomycota* (57.64–91.43%) and *Ascomycota* (6.04–38.52%) across the four replicates. *Rozellomycota* also showed a considerable relative abundance (1.42–3.02%) across all replicates. As shown in Figure 5B. In the SMO20 group, *Basidiomycota* (7.60–84.52%), *Ascomycota* (9.68–44.49%), and *Mortierellomycota* (2.20–41.07%) exhibited notably high relative abundances. In addition, *Glomeromycota* was present at a high relative abundance (0.57–4.33%) in all four replicates. In the SMO30 group (Figure 5C), *Basidiomycota* (66.27–82.19%) and *Ascomycota* (16.19–30.95%) demonstrated extremely high relative abundances across replicates. For the SMO500 group (Figure 5D), the fungal community was characterized by very high relative abundances of *Basidiomycota* (13.99–73.17%), *Ascomycota* (19.31–82.27%), and *Mortierellomycota* (2.20–41.07%). In the SMO650 group (Figure 5E), *Ascomycota* (50.71–75.57%) and *Basidiomycota* (14.09–32.92%) displayed the highest relative abundances. *Mortierellomycota* (1.65–17.44%) and *Chytridiomycota* (1.52–6.57%) were also present at high levels in all four replicates. Overall, the within-group analyses indicated substantial variation in soil fungal community structure across the four replicates within each treatment group. In addition, the same community type showed considerable differences in the relative abundances of fungal taxa across replicates, highlighting notable intra-group heterogeneity.

Figure 5F illustrates the relative abundances of soil fungal communities at the phylum level across the five treatment groups subjected to different mercury contents. *Basidiomycota* (26.34–73.67%) and *Ascomycota* (18.23–58.39%) were the predominant fungal taxa in all groups. *Mortierellomycota* also showed relatively high abundances (1.14–17.36%), particularly in SMO2, SMO20, and SMO650 groups, compared to the SMO30 and SMO500 groups. In contrast, *Chytridiomycota* was notably abundant only in the SMO650 group, with relative abundances ranging from 1.52 to 6.57%. Intergroup analysis revealed that while similar taxonomic groups dominated the fungal communities across the five treatments, their relative abundances varied significantly at the phylum level.

### 3.5 The soil fungal community structure at the class level

**Figure 6:**
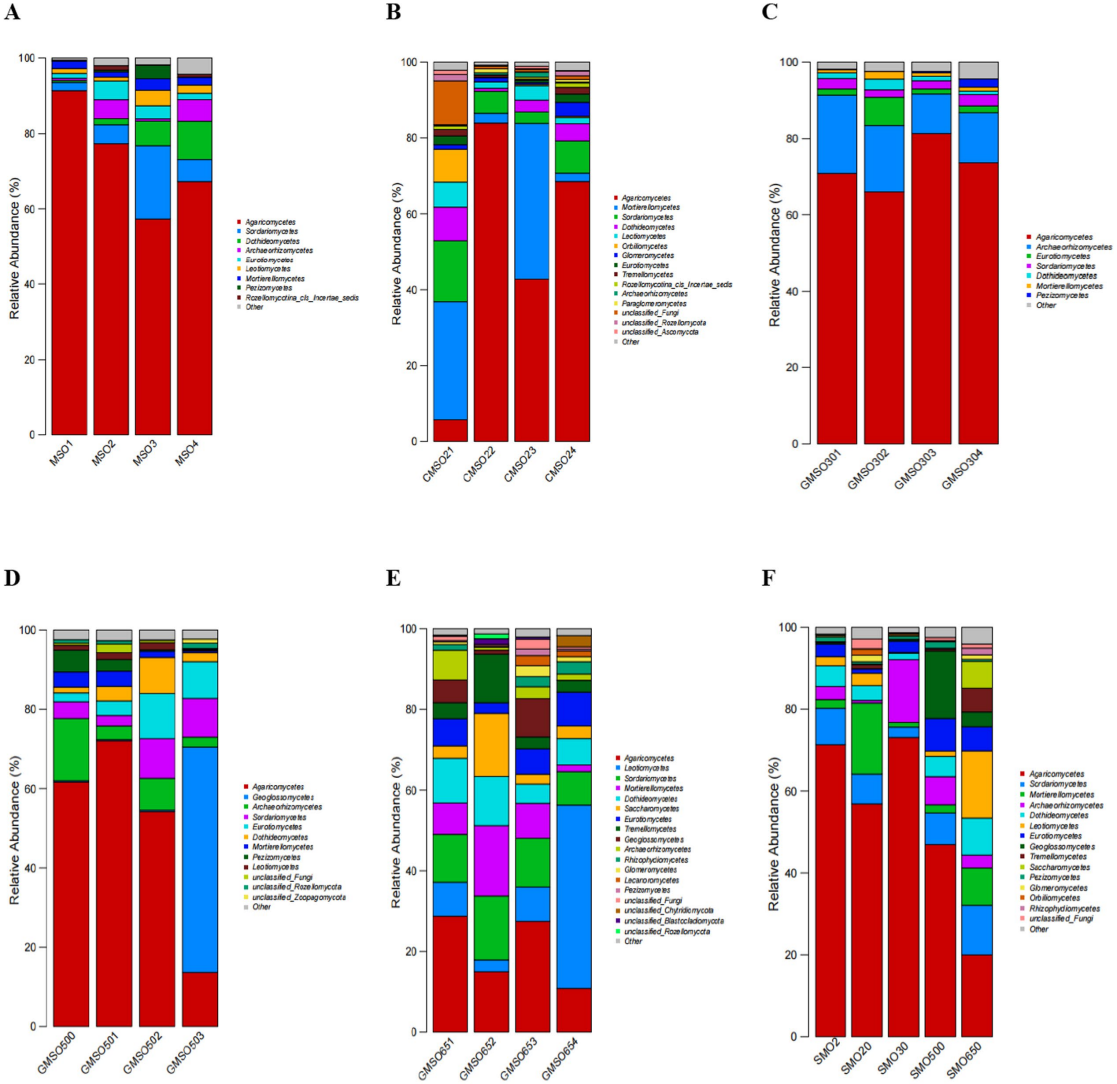
**(A)** The relative abundances of soil fungal community members of the SMO2 group at the class level. **(B)** Relative abundances of soil fungal community members in the SMO20 group at the class level. **(C)** Relative abundances of soil fungal community members in the SMO30 group at the class level. **(D)** Relative abundances of soil fungal community members in the SMO500 group at the class level. **(E)** Relative abundances of soil fungal community members in the SMO650 group at the class level. **(F)** The relative abundances of soil fungal community members of five different groups at the class level.

Figure 6 presents the class-level relative abundances of soil fungal communities under different mercury contents. In the SMO2 group (Figure 6A), *Agaricomycetes* (57.31–91.36%) and *Sordariomycetes* (2.18–19.54%) exhibited the highest relative abundances across replicates. *Dothideomycetes* (1.60–10.30%), *Eurotiomycetes* (1.30–4.95%), and *Mortierellomycetes* (1.42–3.02%) were also consistently abundant. For the SMO20 group (Figure 6B), *Agaricomycetes* (5.72–83.92%), *Mortierellomycetes* (2.20–41.07%), and *Sordariomycetes* (3.01–16.08%) showed notably high relative abundances. *Dothideomycetes* (0.80–8.86%) and *Leotiomycetes* (1.66–6.60%) were also prominently present in all replicates. In the SMO30 group (Figure 6C), *Agaricomycetes* (65.99–81.39%) and *Archaeorhizomycetes* (10.20–20.40%) dominated, with *Eurotiomycetes* (1.32–7.22%) and *Sordariomycetes* (1.96–2.97%) also displaying considerable abundances. The SMO500 community (Figure 6D) was characterized by high abundances of *Agaricomycetes* (13.64–72.08%), *Archaeorhizomycetes* (2.49–15.70%), *Sordariomycetes* (2.64–10.01%), *Eurotiomycetes* (2.25–11.38%), and *Dothideomycetes* (1.47–9.10%), along with *Mortierellomycetes* (0.43–3.85%). Under the high stress condition (Figure 6E), multiple classes showed elevated abundances: *Agaricomycetes* (10.84–28.72%), *Sordariomycetes* (8.28–15.85%), *Leotiomycetes* (2.93–45.44%), *Dothideomycetes* (4.78–12.19%), *Saccharomycetes* (2.43–15.65%), *Mortierellomycetes* (1.66–17.44%), and *Tremellomycetes* (2.93–12.06%). *Archaeorhizomycetes* (0.70–7.38%) were also consistently present. Substantial variations in community structure and taxon abundances were observed among replicates within the same group, particularly in SMO2, SMO20, SMO500, and SMO650 treatments, indicating considerable intra-group heterogeneity.

Figure 6F illustrates the relative abundances of soil fungal communities at the class level across the five treatment groups subjected to different mercury contents. *Agaricomycetes* (19.98–73.09%), *Sordariomycetes* (2.52–12.12%), and *Mortierellomycetes* (4.56–9.86%) showed notably high relative abundances in all groups. Several other classes, *Archaeorhizomycetes* (0.65–15.33%), *Dothideomycetes* (1.54–9.05%), *Leotiomycetes* (0.24–16.39%), and *Eurotiomycetes* (1.09–7.97%) were also consistently abundant across the five treatments. Intergroup analysis indicated that although some taxa were dominant across all groups, the composition of fungal communities varied. Moreover, the relative abundances of the same dominant fungal taxa differed significantly among the five groups, reflecting an influence of mercury content on community structure at the class level.

### 3.6 Relationships between the soil environmental factors and the soil fungal community structure of the five different groups at the phylum and class levels

**Figure 7:**
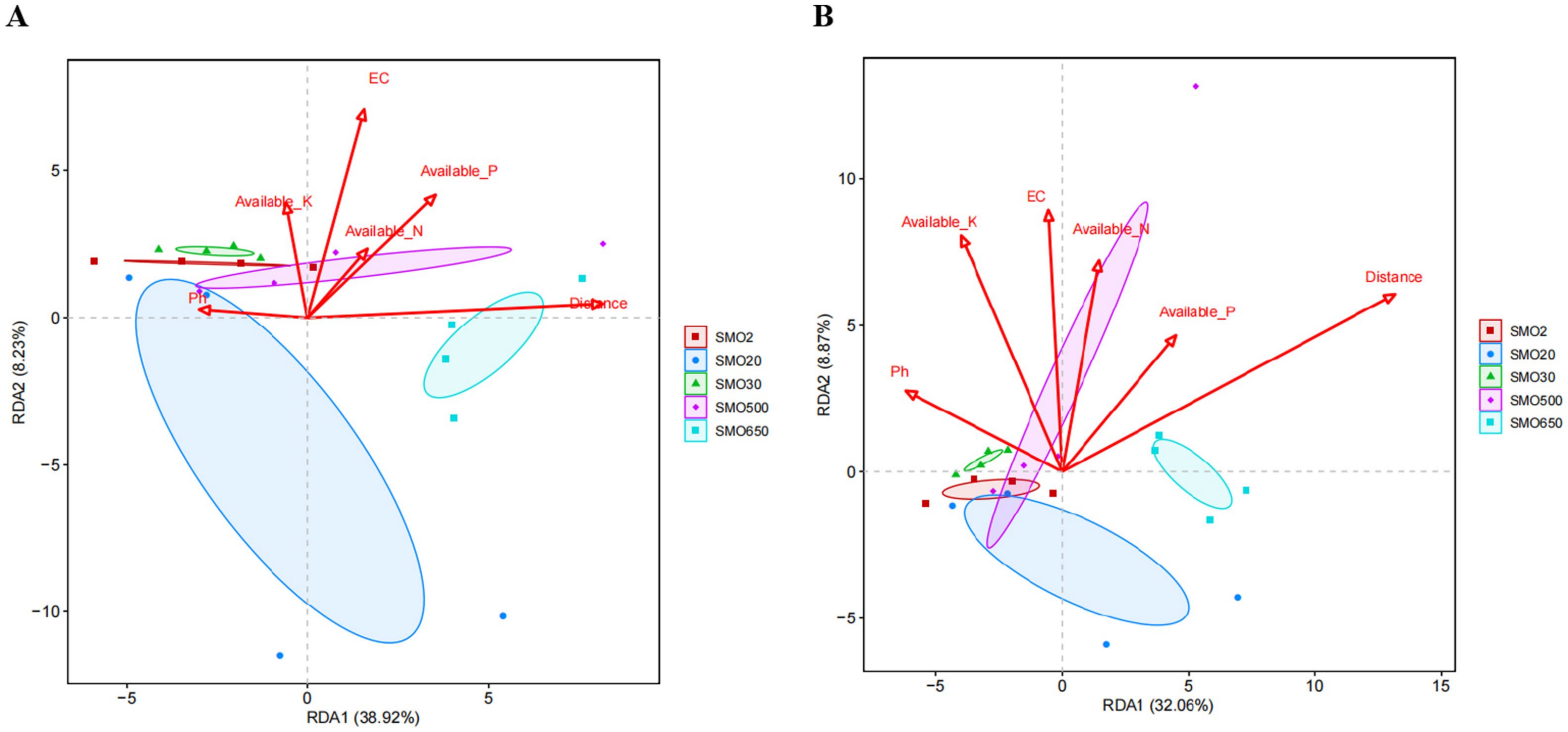
**(A)** The redundancy analysis (RDA) between soil environmental factors and soil fungal community structure for five groups at the phylum level. **(B)** The redundancy analysis (RDA) between soil environmental factors and soil fungal community structure for five groups at the class level (R vegan package (version 2.5–6) analysis).

The redundancy analysis (RDA) results showed that soil environmental factors collectively explained 47.15% of the total variation in soil fungal community structure at the phylum level (Figure 7A) and 40.93% at the class level (Figure 7B). At the phylum level, distance (mercury content), electrical conductivity (EC μs/cm), and available phosphorus (AP) were identified as the main drivers influencing the fungal community composition. At the class level, distance (mercury content), EC, and available potassium (AK) emerged as the dominant factors. At both taxonomic levels, pH exhibited a negative correlation with the other five environmental factors. Overall, mercury content (represented by the distance factor) was the primary driver shaping the soil fungal community structure across the five treatment groups at both the phylum and class levels.

## 4 Discussion

Previous research has consistently demonstrated that mercury and other heavy metals can significantly alter the composition and structure of soil microbial communities (Salam et al., 2020; Wang et al., 2025). Different microbial groups exhibit distinct levels of tolerance to stress, such as cadmium (Pulikova et al., 2025). For example, acid mine drainage from copper mining activities has been shown to adversely affect bacterial communities by restricting microbial metabolic activity and other ecological functions, thereby reducing overall soil microbial diversity (Yin, 2007). Furthermore, the specific types and concentrations of heavy metals present in soil play a critical role in determining the extent and direction of changes in microbial diversity (Montes et al., 2025).

Mercury contamination in soil significantly compromises the stability of soil ecosystems. For instance, it alters the composition and spatial distribution of key soil microorganisms, including bacteria, fungi, and actinomycetes (Frossard et al., 2018; Mi et al., 2023). In this study, an analysis of soil fungal community diversity across five treatment groups subjected to different mercury stress levels near a mercury mining area revealed that, at the phylum level, the total number of OTUs was higher in SMO2, SMO20, and SMO30 groups than in SMO500 and SMO650 groups. At the class level, the SMO2 and SMO30 groups had more OTUs than the SMO20, SMO500, and SMO650 groups. These findings diverge from those reported by Xie et al. (2011), Frossard et al. (2017), and William et al. (2017). We hypothesize that the variations in fungal community diversity among the five treatment groups may be associated with differences in soil environmental factors, such as mercury content, pH, electrical conductivity (EC μs/cm), available nitrogen (AN), available phosphorus (AP), and available potassium (AK). Notably, the environmental conditions in this study differed substantially from those in many earlier experiments, which were often conducted under artificially simulated mercury stress (Xie et al., 2011; Feng et al., 2024; Harris-Hellal et al., 2009; Wang, 2017). The naturally heterogeneous soil environment at our sampling site, a mercury-mining area without exogenous manipulation, likely contributed significantly to the observed fungal community diversity. This may explain why our results align more closely with studies conducted in similar natural settings, such as those by Frossard et al. (2017) and Mi et al. (2023). A common finding across these studies is that high soil mercury content significantly reduces fungal community diversity. The survival of mercury-resistant fungal taxa represents an adaptive strategy that ensures evolutionary resilience in polluted soils. The survival of dominant fungal communities under high mercury stress provides strong evidence of fungal community adaptation to long-term mercury-contaminated environments. This observation is consistent with the findings of Yan et al. (2018), who demonstrated that severe heavy metal pollution can promote the diversity of mercury-resistant fungal taxa while reducing the diversity of non-tolerant fungal taxa. Furthermore, fungal community diversity does not follow a clear mercury gradient, which we preliminarily attribute to soil heterogeneity and the adaptive evolution of fungal communities.

We assessed the alpha diversity of fungal communities using several metrics: the number of sequencing reads, the count of operational taxonomic units (OTUs), and the Shannon and Simpson indices. Significant differences were observed across the five treatment groups in terms of reads, OTUs, and the Shannon index, whereas the Simpson index did not show significant variation. A comprehensive comparison based on four indicators revealed the following order of fungal community diversity: SMO500 > SMO2 > SMO30 > SMO20 > SMO650. These findings indicate that fungal community diversity is influenced by various intrinsic factors in the natural soil environment. Variation in these underlying environmental conditions contributed to distinct fungal community profiles among the five groups, underscoring considerable internal heterogeneity within each treatment group.

Soil microbial community structure serves as a key indicator of the structural integrity and stability of soil ecosystems, and it can effectively reflect the degree of soil environmental pollution (Zhang et al., 2022). As crucial components of soil microbiota, fungi are involved in organic matter decomposition, drive nutrient cycling, supply plants with essential nutrients, and contribute significantly to soil ecosystem health (Hu et al., 2018). The composition and abundance of soil fungal communities are influenced by a range of environmental factors, including soil nutrient status, aeration, pH, and heavy metal concentrations (Du et al., 2023; Yan et al., 2018). In this study, we examined the distribution and composition of dominant fungal taxa within and across five groups. The results revealed that the dominant taxa, both those shared and those unique to different groups, varied in composition at both the phylum and class levels. At the phylum level, *Basidiomycota* (26.34–73.67%), *Ascomycota* (18.23–58.39%), and *Mortierellomycota* (1.14–17.36%) were the most representative fungal groups across all five treatments. This finding aligns with previously reported effects of cadmium pollution on fungal communities (Hu et al., 2018). At the class level, *Agaricomycetes* (19.98–73.09%), *Sordariomycetes* (2.52–12.12%), and *Mortierellomycetes* (4.56–9.86%) were the dominant taxa, while *Archaeorhizomycetes* (0.65–15.33%), *Dothideomycetes* (1.54–9.05%), *Leotiomycetes* (0.24–16.39%), and *Eurotiomycetes* (1.09–7.97%) also showed considerable presence across the five groups. Our findings indicate that both common and unique fungal taxa dominated across the five groups, and their relative abundances varied not only among replicates within the same group but also across different treatment groups. These differences may be attributed to variations in mercury content, soil type, and fundamental soil properties, which collectively influence mercury bioavailability and toxicity. The high variability in fungal taxa among replicates suggests that field soil is inherently heterogeneous. This variability in fungal taxa among replicates is a key characteristic of field research; it is a direct manifestation of the system’s true complexity rather than an artifact observed in controlled experiments with homogenized soil. The persistence of these common and unique fungal taxa under high mercury stress suggests a strong selective pressure for mercury resistance and enhanced adaptability to other soil environmental factors (Frossard et al., 2018; Alexis et al., 2020). Furthermore, the variation in the relative abundances of these dominant taxa within and across groups appears to be closely linked to the intrinsic heterogeneity of mercury-contaminated soils (Dranguet et al., 2019; Osterwalder et al., 2019; Zhu et al., 2021).

Soil environmental factors, including pH, EC (μs/cm), nutrient availability, and heavy metal content, are known to influence the structure of soil microbial communities (Mija et al., 2021; Mbuthia et al., 2015). As a significant environmental stressor, mercury pollution has been widely observed to reduce soil fungal diversity and alter community composition. In this study, redundancy analysis (RDA) revealed that mercury content (represented by the distance factor), pH, EC (μs/cm), available phosphorus (AP), available potassium (AK), and available nitrogen (AN) were all associated with the distribution of soil fungal communities at both the phylum and class levels. This result indicated a strong correlation between fungal communities and soil environmental conditions in the study area. Among these factors, mercury content demonstrated a predominant influence in shaping fungal community diversity and structure in the studied terrestrial ecosystem. This finding aligns with the results reported by Alexis et al. (2020) but contrasts with those of Huang et al. (2024), which emphasized soil pH as the strongest driver of fungal beta diversity, surpassing the effects of climate, soil nutrients, and plant properties.

To isolate fungal communities resistant to mercury from these highly adaptive communities, selection and breeding should prioritize strains capable of nitrogen fixation, phosphorus solubilization, or heavy-metal passivation for use as soil remediation agents. Promising strains can be further encapsulated using alginate or chitosan-based microencapsulation to create sustained-release formulations. This approach significantly improves the survival of mercury-resistant fungal communities under harsh conditions and enables controlled release, thereby enhancing long-term remediation efficacy and ecological adaptability. The development of such heavy-metal-passivating fungal strains will help reduce heavy-metal accumulation in tea and other plants, thereby lowering associated safety risks. This strategy shows substantial potential to support vegetation restoration in mercury-impacted areas and to facilitate non-food crop cultivation, which represent key focuses of our future research.

## 5 Conclusion

Based on the above investigation into the effects of mercury pollution on soil fungal communities in naturally long-term contaminated environments, we draw the following conclusions:

(1) At both the phylum and class levels, soil fungal community diversity was greater in the SMO2 and SMO30 groups than in the SMO20, SMO500, and SMO650 groups. Furthermore, the diversity across the five soil sample groups did not exhibit a consistent trend with increasing mercury concentration.
(2) The same dominant fungal taxa were present within each group and across the five different groups, yet their relative abundances varied considerably both within the same group and among the different groups at both taxonomic levels.
(3) The diversity and structure of the soil fungal community were strongly associated with soil internal environmental factors at the phylum and class levels. Among these factors, mercury content played a paramount role in shaping fungal community composition and diversity.

## Data Availability

The datasets presented in this study can be found in online repositories. The names of the repository/repositories and accession number(s) can be found at: https://ngdc.cncb.ac.cn/gsa/browse/CRA011955, CRA011955.
