# Supplementary figures and images for: Response of soil fungal communities to long-term soil mercury pollution
Source: Front Microbiol. 2026 Jun 10;17:1827403. doi: 10.3389/fmicb.2026.1827403 (PMC13290816; doi:10.3389/fmicb.2026.1827403)

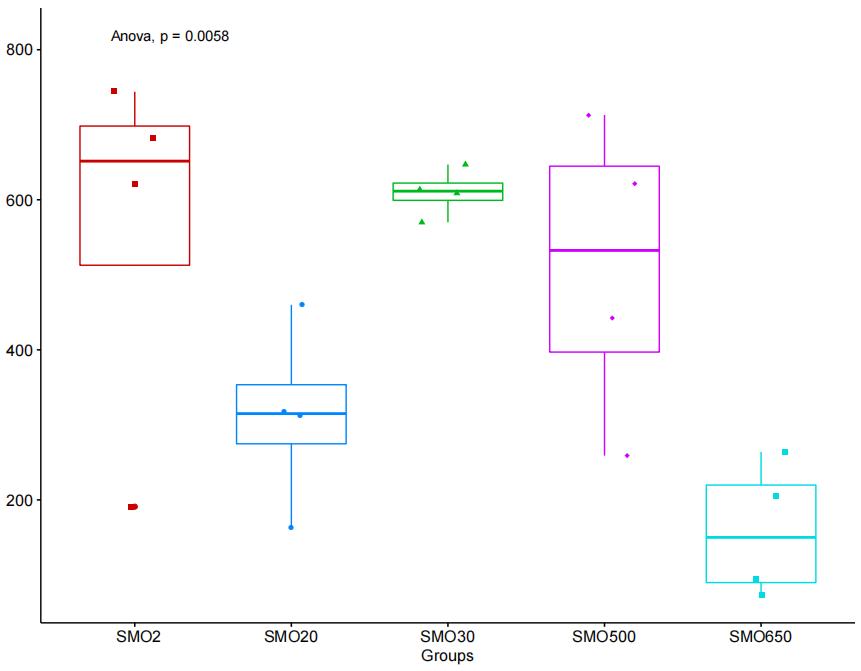

Supplement: Supplementary file 1 [file Image_1.JPEG]

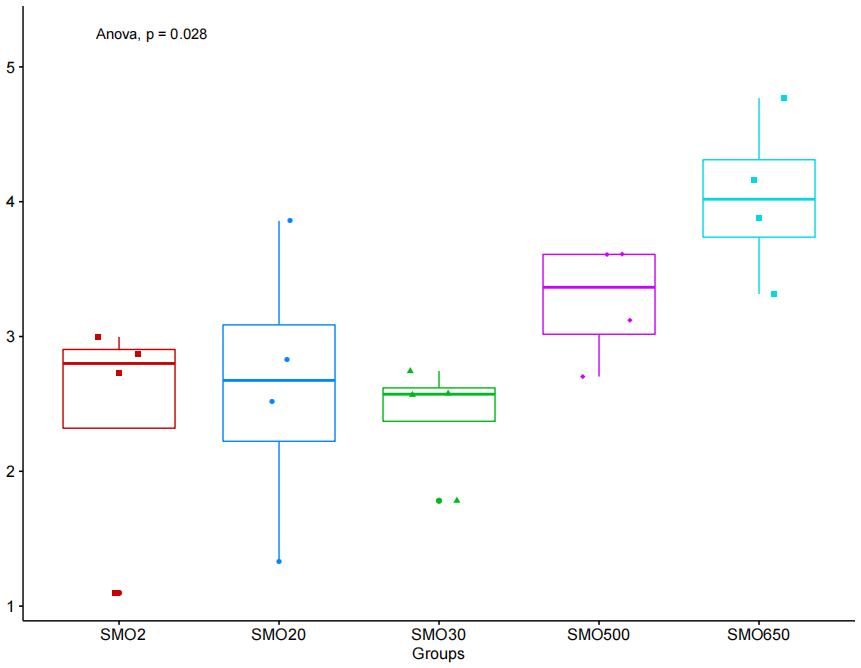

Supplement: Supplementary file 2 [file Image_2.JPEG]

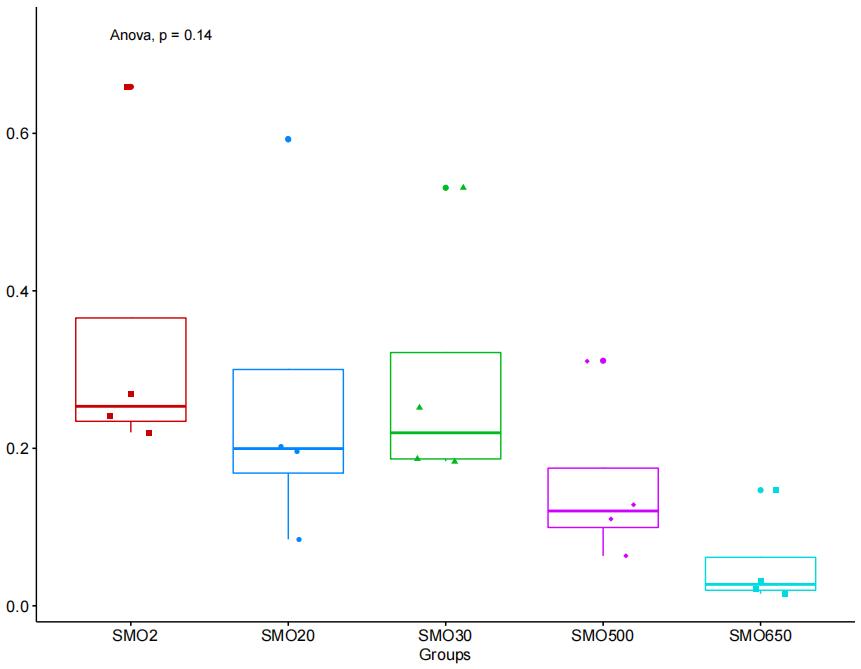

Supplement: Supplementary file 3 [file Image_3.JPEG]

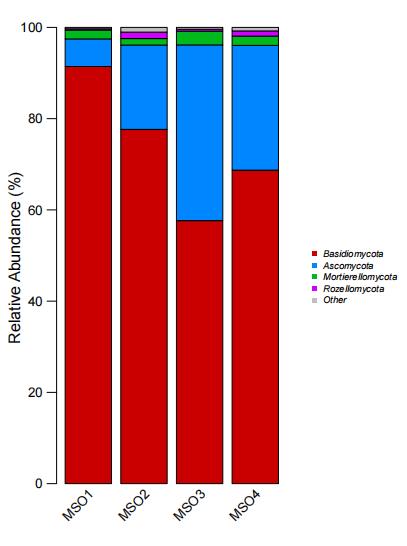

Supplement: Supplementary file 4 [file Image_4.JPEG]

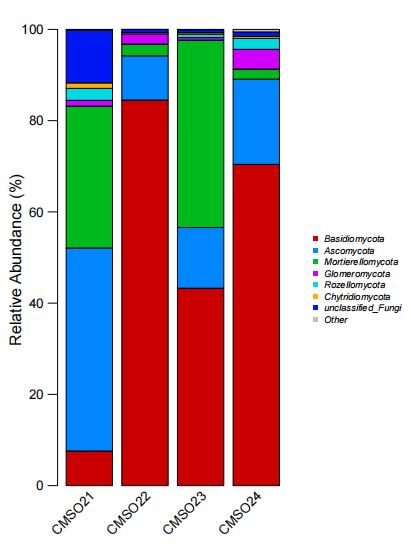

Supplement: Supplementary file 5 [file Image_5.JPEG]

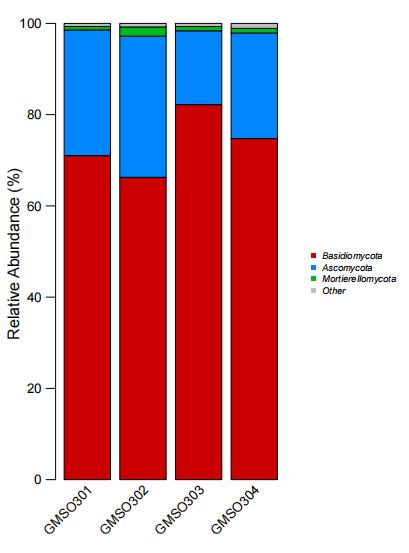

Supplement: Supplementary file 6 [file Image_6.JPEG]

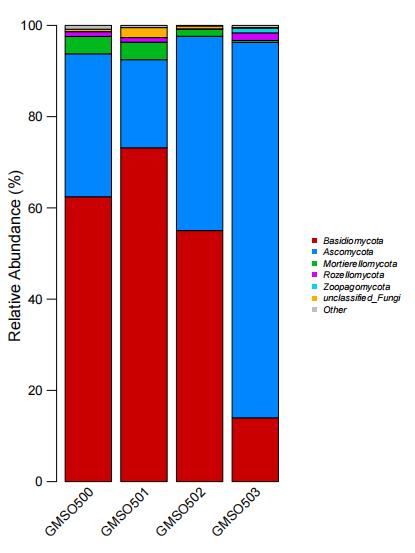

Supplement: Supplementary file 7 [file Image_7.JPEG]

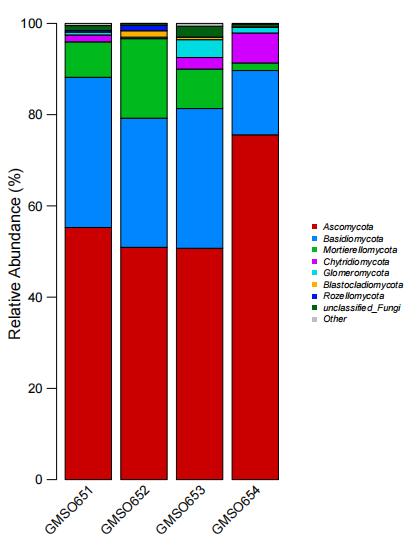

Supplement: Supplementary file 8 [file Image_8.JPEG]

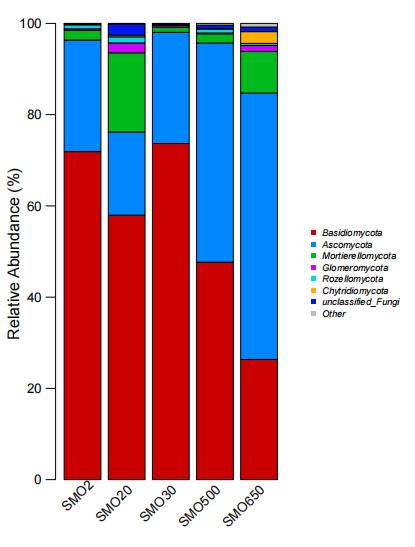

Supplement: Supplementary file 9 [file Image_9.JPEG]

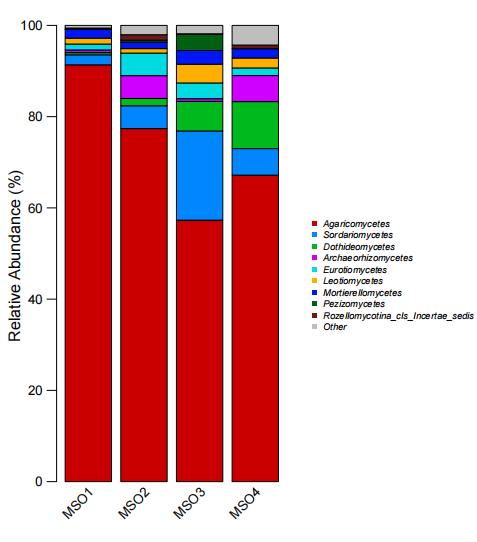

Supplement: Supplementary file 10 [file Image_10.JPEG]

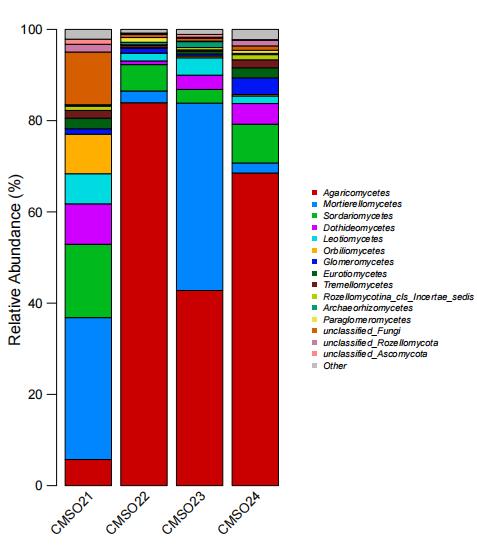

Supplement: Supplementary file 11 [file Image_11.JPEG]

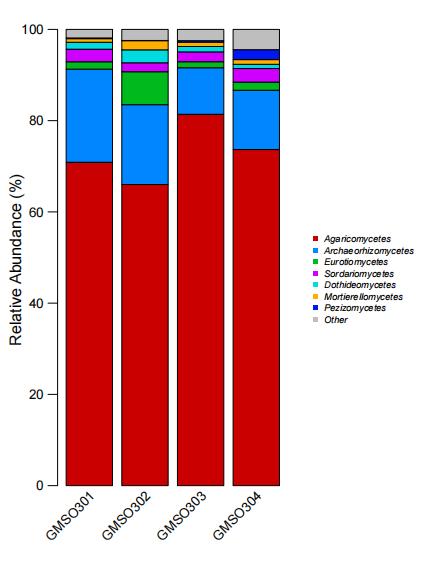

Supplement: Supplementary file 12 [file Image_12.JPEG]

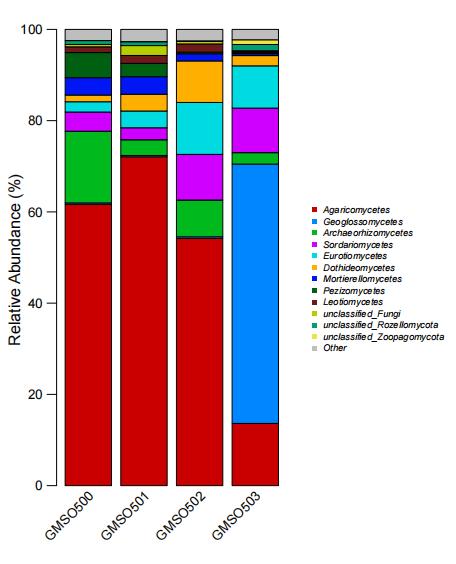

Supplement: Supplementary file 13 [file Image_13.JPEG]

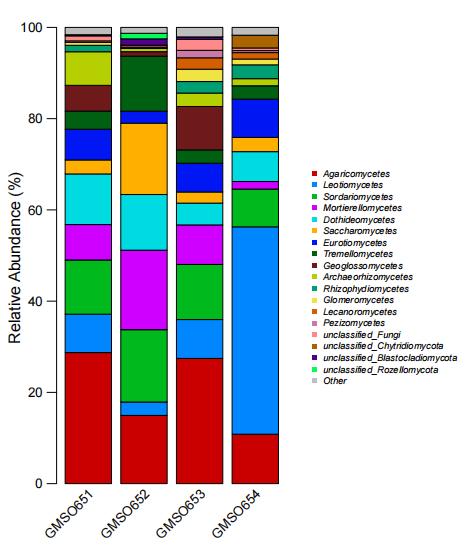

Supplement: Supplementary file 14 [file Image_14.JPEG]

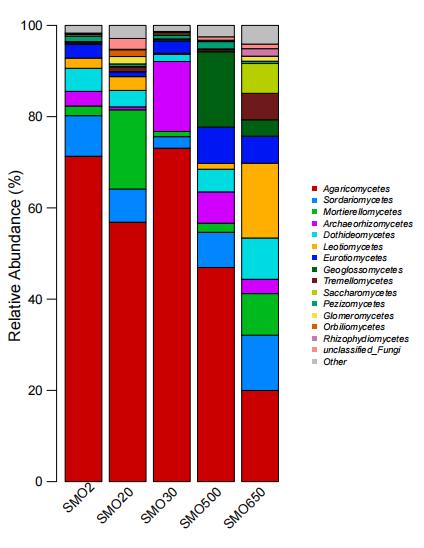

Supplement: Supplementary file 15 [file Image_15.JPEG]
